# S100β-Positive Cells of Mesenchymal Origin Reside in the Anterior Lobe of the Embryonic Pituitary Gland

**DOI:** 10.1371/journal.pone.0163981

**Published:** 2016-10-03

**Authors:** Kotaro Horiguchi, Hideji Yako, Saishu Yoshida, Ken Fujiwara, Takehiro Tsukada, Naoko Kanno, Hiroki Ueharu, Hiroto Nishihara, Takako Kato, Takashi Yashiro, Yukio Kato

**Affiliations:** 1 Laboratory of Anatomy and Cell Biology, Department of Health Sciences, Kyorin University, Mitaka, Tokyo, Japan; 2 Institute of Endocrinology, Meiji University, Kawasaki, Kanagawa, Japan; 3 Division of Life Science, Graduate School of Agriculture, Meiji University, Kawasaki, Kanagawa, Japan; 4 Division of Histology and Cell Biology, Department of Anatomy, Jichi Medical University School of Medicine, Shimotsuke, Tochigi, Japan; Temple University School of Medicine, UNITED STATES

## Abstract

The anterior and intermediate lobes of the pituitary gland develop through invagination of the oral ectoderm and as they are endocrine tissues, they participate in the maintenance of vital functions via the synthesis and secretion of numerous hormones. We recently observed that several extrapituitary cells invade the anterior lobe of the developing pituitary gland. This raised the question of the origin(s) of these S100β-positive cells, which are not classic endocrine cells but instead comprise a heterogeneous cell population with plural roles, especially as stem/progenitor cells. To better understand the roles of these S100β-positive cells, we performed immunohistochemical analysis using several markers in S100β/GFP-TG rats, which express GFP in *S100β*-expressing cells under control of the *S100β* promoter. GFP-positive cells were present as mesenchymal cells surrounding the developing pituitary gland and at Atwell's recess but were not present in the anterior lobe on embryonic day 15.5. These cells were negative for SOX2, a pituitary stem/progenitor marker, and PRRX1, a mesenchyme and pituitary stem/progenitor marker. However, three days later, GFP-positive and PRRX1-positive (but SOX2-negative) cells were observed in the parenchyma of the anterior lobe. Furthermore, some GFP-positive cells were positive for vimentin, p75, isolectin B4, DESMIN, and Ki67. These data suggest that S100β-positive cells of extrapituitary origin invade the anterior lobe, undergoing proliferation and diverse transformation during pituitary organogenesis.

## Introduction

The adenohypophysis, which is composed of anterior and intermediate lobes, develops through invagination of the oral ectoderm under the influence of several growth factors by contacting the diencephalon and both sides of the ectoderm [1–3]. Both the anterior and intermediate lobes contain six types of differentiated cells that play important roles in the synthesis and secretion of several hormones. These endocrine cells are required in all vertebrates for the maintenance of vital functions such as reproduction, metabolism, growth, and homeostasis. Additionally, substantial populations of non-hormone-producing cells exist in the anterior and intermediate lobes and participate in maintaining, assisting, and supplementing hormone-producing cells and the vessel system. For quite some time, the non-endocrine cells that have attracted the most attention are folliculo-stellate (FS) cells, which have a star-like shape [4]. S100β, a Ca^2+^-binding protein, is a marker for FS cells. S100β-positive cells in the anterior lobe are believed to have several roles, acting as stem cells, phagocytes, cells that regulate hormone release, and cells that participate in cell-cell communication [5–7].

Recently accumulated data indicate that S100β-positive cells are composed of heterogeneous cell populations that are relevant to several functions. Immunohistochemical analysis with stem/progenitor cell markers revealed that S100β-positive cells are composed of at least three groups of cells [8]. S100β-positive cells can also be grouped into two cell types based on their adhesiveness to the extracellular matrix: stellate-shaped cells and dendritic-like cells [9]. As postulated previously, some S100β-positive cells have the ability to differentiate into skeletal muscle cells [10–12]. More recently, we have reported that some S100β-positive cells are able to differentiate into all hormone-producing cell types in the anterior and intermediate lobes [13]. Despite these new findings, it is not yet clear how S100β-positive cells originate and develop into plural states with diverse roles.

Facilitating further investigation of the roles of S100β-positive cells, a transgenic rat that expresses green fluorescent protein (GFP) under the control of the *S100β* promoter (S100β/GFP-TG rat) has been generated [14]. Using the S100β/GFP-TG rat, we observed that *S100β* transcripts are present in the embryonic pituitary on embryonic day 21.5 (E21.5) [8], though it was previously believed that S100β-positive cells do not appear until approximately ten days after birth [15]. In the present study, we examined the appearance of S100β-positive cells in the embryonic pituitary and their characteristics via immunohistochemistry using several marker proteins. As a result, we observed that S100β/GFP-positive cells are present in the prenatal pituitary, appearing by migration from Atwell's recess, an intraglandular fossa that receives several blood vessels [16]. These cells are present with mesenchymal cells and other cell types that surround the pituitary gland. They exhibit proliferative activity and co-expression with several markers of vessels or neural crest cells, and they reflect transient, multipotent, and migratory characteristics. Thus, our results suggest that some S100β-positive cells are extrapituitary in origin and partially participate in vasculogenesis and formation of the pituitary gland.

## Materials and Methods

### Ethic Statement

All animal experiments were performed following approval from the Institutional Animal Experiment Committee of Meiji University (IACUC 14–0012) and Jichi Medical University (No. 13004 and 14051) and were conducted in accordance with the Institutional Regulations of Animal Experiments and Fundamental Guidelines for Proper Conduct of Animal Experiments and Related Activities in Academic Research Institutions under the jurisdiction of the Japanese Ministry of Education, Culture, Sports, Science and Technology. All treatments were performed under deep anesthesia and all efforts were made to minimize suffering. All rats did not become severely ill or died at any time prior to the experimental endpoint. Rats were sacrificed by exsanguination from the right atrium under deep pentobarbital anesthesia (40mg/kg) and then perfused with 4% paraformaldehyde in 0.05 M phosphate buffer (pH 7.4) for experiments.

### Rats

S100β/GFP-TG rats [14] that express GFP under control of the promoter for the *S100β*gene, a marker of FS cells, were provided by Professor K. Inoue of Saitama University and bred in our laboratory. Male rats 8–10 weeks old weighing 250–300 g were provided with *ad libitum* access to food and water and housed under conditions of 12 h light and 12 h darkness.

### Immunohistochemistry

Heads of S100β/GFP-TG embryonic rats on E15.5, E18.5, E19.5, and E20.5 were fixed in 4% paraformaldehyde buffered with 0.05 M phosphate buffer (PB; pH 7.4) for 20–24 h at 4°C, followed by immersion for more than two days in PB (pH 7.2) containing 30% sucrose at 4°C. This was followed by embedding the samples in optimum cutting temperature (O.C.T.) compound (Sakura Finetek Japan, Tokyo, Japan) at -80°C before sectioning. Frozen sections 10 μm thick in the sagittal plane were mounted on glass slides (Matsunami, Osaka, Japan). Antigen retrieval was performed with an Immunosaver (Nisshin EM, Tokyo, Japan) for 60 min at 80°C. Slides were then washed in 20 mM 2-[4-(2-hydroxyethyl)piperazin-1-yl] ethanesulfonic acid (pH 7.5) containing 100 mM NaCl (HEPES), followed by blocking in HEPES containing 0.4% Triton X100 and 10% fetal bovine serum (FBS) or 1% bovine serum albumin (BSA). Primary antibodies, antisera, and lectin were reacted overnight at 4°C. The primary antibodies used were chicken immunoglobulin (Ig) Y against GFP (1:250 dilution, Aves Labs, Inc., Tigard, OR, USA), rabbit IgG against cow S100β (1:1000 dilution, Dako, Glostrup, Denmark), rabbit antiserum against rat PRRX1 (1:1,000 dilution, raised in our laboratory and assessed for specificity) [17], rabbit antiserum against rat PRRX2 (1:1,000 dilution, raised in our laboratory and assessed for specificity) [17], goat IgG against stem/progenitor cell marker human SOX2 (1:500 dilution, Neuromics, Edina, MN, USA), mouse monoclonal antibody against neural crest cell marker rat p75 (1:100 dilution, Abcom, Plc., Cambridge, UK), mouse monoclonal antibody against smooth muscle cell marker human α-SMA (1:100 dilution, Santa Cruz Biotechnology, Santa Cruz, CA, USA), mouse antiserum against pericyte and a neural and mesenchymal stem/progenitor cell marker rat NESTIN (1:250 dilution, BD Biosciences, San Jose, CA, USA), mouse monoclonal antibody against mesenchymal cell marker pig VIMENTIN (1:10,000 dilution, Sigma-Aldrich Corp., St. Louis, MO, USA) and rabbit antibody against dividing cell marker human Ki67 (1:500 dilution, Abcom, Plc.). Afterwards, a cocktail of the following guinea pig antisera against pituitary hormones was used: anti-rat LHβ (1:8,000 dilution), anti-rat FSHβ (1:4,000 dilution), anti-rat TSHβ (1:16,000 dilution), and anti-rat PRL (1:4,000 dilution). The antisera were provided by the National Institute of Diabetes and Digestive and Kidney Disease, courtesy of Dr. A. F. Parlow. Guinea pig anti-human ACTH (1:8,000 dilution) and anti-human GH (1:1,000 dilution) antisera were provided by Dr. S. Tanaka, Shizuoka University, and isolectin B4-conjugated Dyelight 649 (1:100 dilution) was provided by Vector Laboratories (Burlingame, CA, USA). Sections were washed with HEPES, followed by incubation with secondary antibodies, performed with Cy3- or Cy5-conjugated AffiniPure donkey with anti-goat, -mouse, -rabbit, and -guinea pig IgG and fluorescein isothiocyanate (FITC)-conjugated AffiniPure donkey with anti-chicken IgY (1:500 dilution, Jackson ImmunoResearch, West Grove, PA, USA). The sections were again washed with HEPES and then enclosed in VECTASHIELD Mounting Medium with 4',6-diamino-2-phenylindole (DAPI; Vector Laboratories) to stain nuclei. Immunofluorescence was observed under fluorescence microscopy with a BZ-9000 (KEYENCE, Osaka, Japan) and fluorescence confocal microscopy with a FV1000 (OLYMPUS, Tokyo, Japan).

## Results

### Appearance of GFP-positive cells at Atwell's recess on E15.5

**Fig 1:**
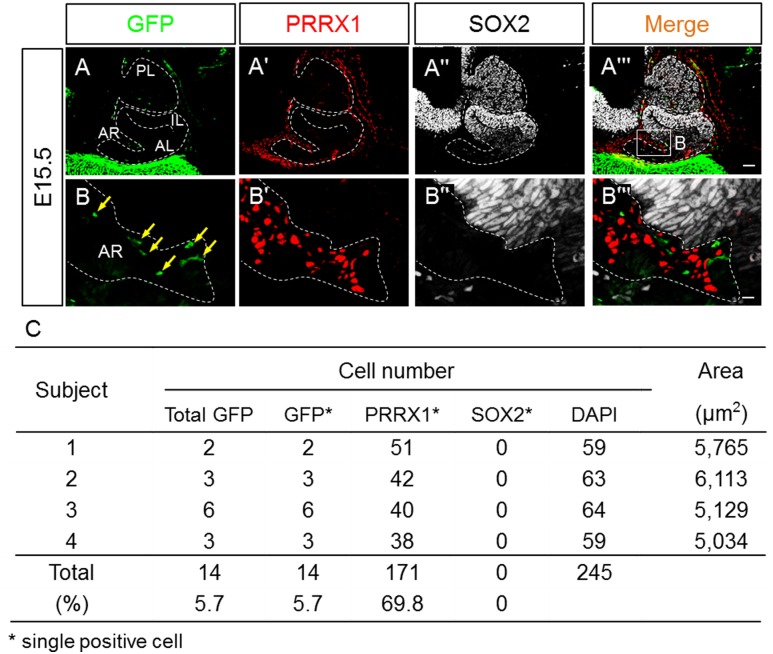
GFP-positive cells at Atwell's recess on E15.5. Using sagittal sections of embryonic pituitaries on E15.5, immunostaining was performed for GFP, PRRX1, and SOX2, which were visualized with FITC (**A** and **B**, *green*), Cy3 (**Aʹ** and **Bʹ**, *red*), and Cy5 (**Aʹʹ** and **Bʹʹ**, *white*), respectively. Cells positive for GFP only are indicated with yellow arrows. The boxed area in **Aʹʹʹ** is enlarged in **B**–**Bʹʹʹ**. Each cell type was counted in four independent pituitaries (n = 4), and results are listed in **C**. *AL* anterior lobe; *IL* intermediate lobe; *PL* posterior lobe; *AR* Atwell's recess. Bars = 50 μm (**A–Aʹʹʹ**) and 10 μm (**B–Bʹʹʹ**).

We first examined whether GFP-positive cells were present in the embryonic pituitary on E15.5 by staining for GFP. As shown in Fig 1, GFP-positive cells were observed at Atwell’s recess, while very strong GFP signals were observed beneath the pituitary gland (Fig 1Aʹ). The recess is characterized as an intraglandular fossa that receives several blood vessels [16]; we have previously suggested that PRRX1-positive cells are present here and invade in order to participate in pituitary vasculogenesis [18,19]. To further characterize the GFP-positive cells, we performed triple immunostaining for GFP, PRRX1, and SOX2. In the enlarged images (Fig 1B–1Bʹʹʹ), it is clear that GFP-positive and PRRX1-positive cells do not overlap, while SOX2-positive cells were not present at the recess, in the brain, or in the anterior pituitary gland (Fig 1Aʹʹ–1Bʹʹ). We analyzed the ratios of GFP- and PRRX1-positive cells to the total number of cells in Atwell’s recess, counted by DAPI staining. GFP-positive cells accounted for approximately 5.7% of cells, while PRRX1-positive cells accounted for 69.8% (Fig 1C).

### GFP-positive cells during pituitary development

**Fig 2:**
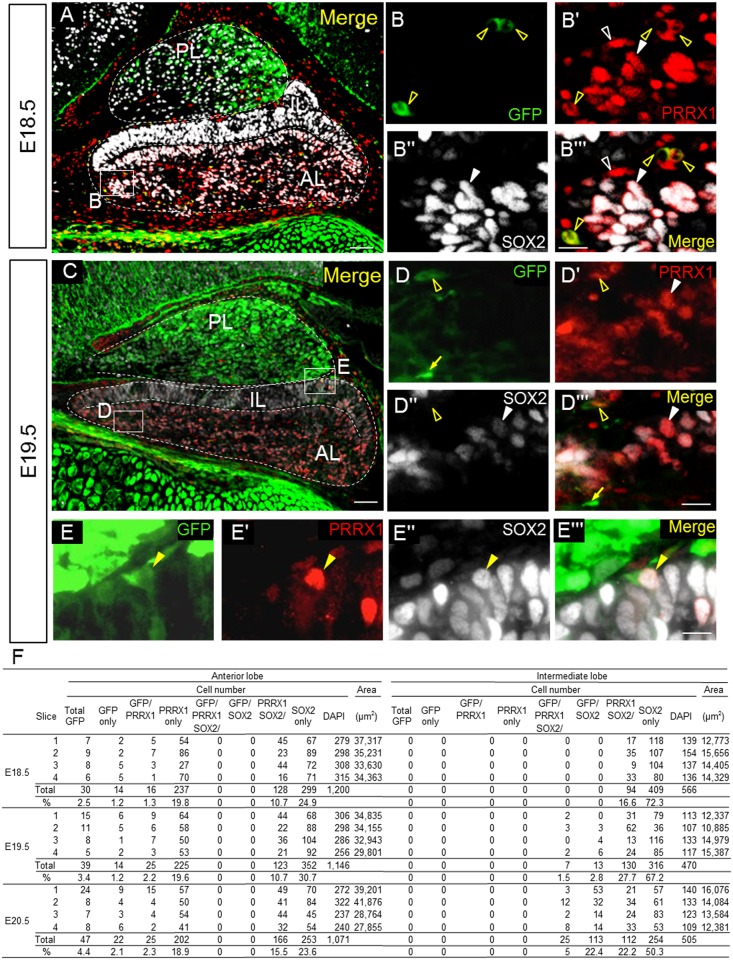
GFP-positive cells during pituitary development. Using sagittal sections of embryonic pituitaries on E18.5 (**A**–**Bʹʹʹ**) and E19.5 (**C**–**Eʹʹʹ**), immunostaining was performed for GFP, PRRX1, and SOX2, which were visualized with FITC (*green*), Cy3 (*red*), and Cy5 (*white*), respectively. GFP/PRRX1/SOX2-triple (*yellow arrowheads*), GFP/PRRX1-double (*yellow open arrowheads*), PRRX1/SOX2-double (*white arrowheads*), GFP-single (*yellow arrows*) and PRRX1-single (*white open arrowheads*) positive cells are indicated. The boxed areas in **A** and **C** are enlarged in **B**–**Bʹʹʹ**, **D**–**Dʹʹʹ**, and **E**–**Eʹʹʹ**. Each cell type was counted (n = 1 for E18.5, n = 2 for E19.5, and n = 2 for E20.5, with four slices each), and results are shown in **F**. *AL* anterior lobe; *IL* intermediate lobe; *PL* posterior lobe. Bars = 50 μm (**A**, **C**) and 10 μm (**B–Bʹʹʹ** and **D–Eʹʹʹ**).

We performed the same histochemical analysis for the late embryonic stages (Fig 2). Enlarged images of the rostral part of the anterior pituitary on E18.5 and E19.5 reveal the presence of GFP-single (Fig 2D–2Dʹʹʹ, yellow arrow), PRRX1-single (Fig 2B–2Bʹʹʹ, white open arrowhead), and GFP/PRRX1-double positive cells (Fig 2B–2Bʹʹʹ and 2D–2Dʹʹʹ, yellow open arrowheads). Cells were counted in the anterior and intermediate lobes of four sections each on E18.5, E19.5, and E20.5 (image data not shown), as listed in Fig 2F. Results showed that in the anterior lobe, GFP-single and GFP/PRRX1-double positive cells were present at low frequencies (1.2–2.3%), while PRRX1-single positive cells were more prevalent (18.9–19.8%; Fig 2F). SOX2-positive cells were negative for GFP, and the frequency of SOX2/PRRX1-double cells was 10.7–15.5% (Fig 2F). In the intermediate lobe, GFP/SOX2/PRRX1-triple positive cells (Fig 2E–2Eʹʹʹ) were observed, but GFP- and/or PRRX1-single and -double positive cells were absent on E19.5 and E20.5 (Fig 2F).

We have previously shown that PRRX2, a cognate of PRRX1, is expressed in the mesenchyme cells surrounding the pituitary gland [19]. Triple immunostaining for GFP, SOX2, and PRRX2 was performed on E20.5. As shown in S1 Fig, PRRX2-positive cells were not observed in the pituitary gland, but they were present in the surrounding mesenchyme, especially outside the posterior lobe. GFP/PRRX2-double positive cells, as well as GFP- and PRRX2-single positive cells (S1B–S1Bʹʹʹ Fig), were observed in the caudal and dorsal area outside of the pituitary. As PRRX2 was not present in the pituitary, we did not include PRRX2 in further staining experiments.

### Proliferative ability of GFP-positive cells

**Fig 3:**
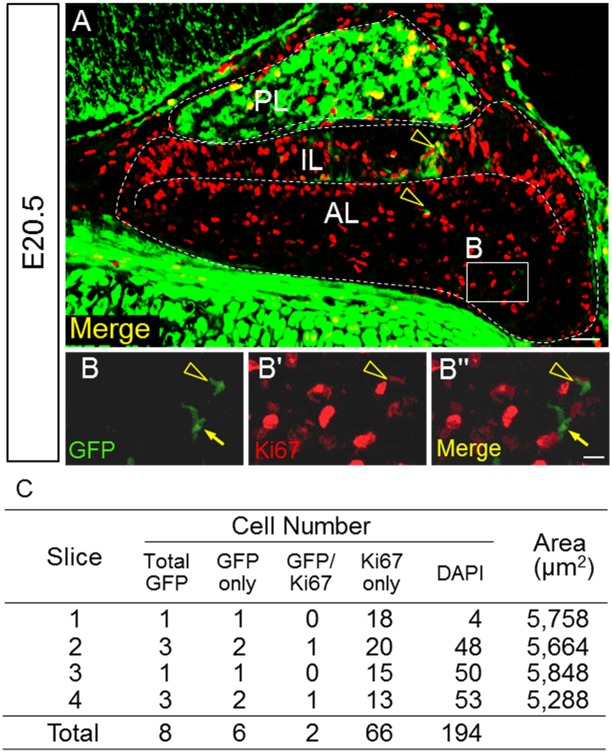
Double immunostaining for GFP and Ki67. Double immunostaining for GFP (*green*) and Ki67 (*red*) was performed on a section on E20.5. The boxed area in **A** is enlarged in **B**–**Bʹʹ**. GFP/Ki67-double positive (*yellow open arrowheads*) and GFP-single positive (*yellow arrow*) cells are indicated. Each cell type was counted in the anterior lobe (n = 2, with one slice each), and results are shown in **C.** *AL* anterior lobe; *IL* intermediate lobe; *PL* posterior lobe. Bars = 50 μm (**A**) and 10 μm (**B–Bʹʹ**).

Immunohistochemical analysis of Ki67, a cell division marker, was performed together with GFP staining to verify the proliferative activity of GFP-positive cells on E20.5. There were GFP-positive cells that were also positive for Ki67, and these were small and elongated (Fig 3). In addition, a number of GFP-positive cells in the intermediate lobe were obviously positive for Ki67. A count of the number of immunopositive cells in the anterior lobe showed that a quarter of GFP-positive cells were also positive for Ki67 (Fig 3C).

### Absence of pituitary hormones in GFP-positive cells

**Fig 4:**
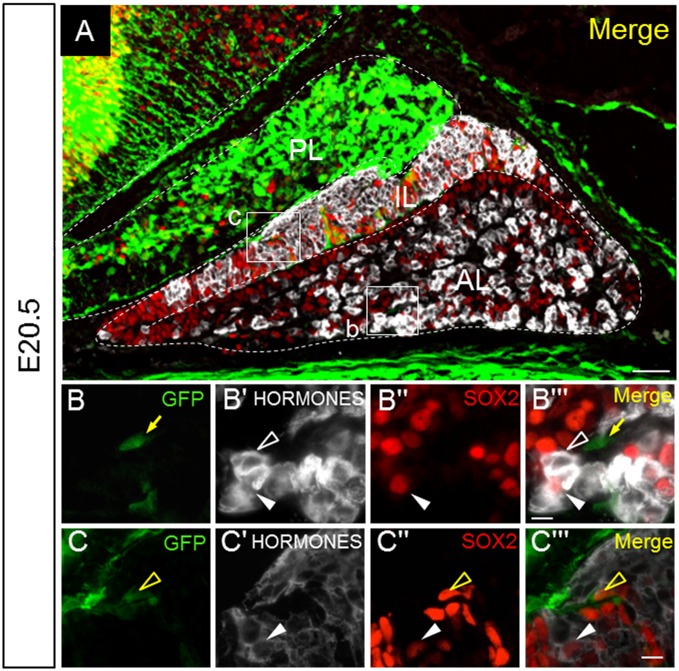
Triple immunostaining for GFP, pituitary hormones, and SOX2. Triple immunostaining using a section from E20.5 was performed for GFP (*green*), SOX2 (*red*), and pituitary hormones (*white*) using a cocktail of antibodies against the hormones FSHβ, LHβ, prolactin, TSHβ, ACTH, and GH (HORMONES). The boxed area in **A** is enlarged in **B**–**Bʹʹʹ** and **C**–**Cʹʹʹ**. GFP/SOX2-double positive (*yellow open arrowheads*), SOX2/HORMONES-double positive (*white arrowheads*), GFP-single positive (*yellow arrows*), and HORMONES-single positive (*white open arrowheads*) cells are indicated. *AL* anterior lobe; *IL* intermediate lobe; *PL* posterior lobe. Bars = 50 μm (**A**) and 10 μm (**B**–**Cʹʹʹ**).

We recently demonstrated that a subset of S100β-positive cells prepared from adult rat anterior lobes differentiate into hormone-producing cells [13]. To examine whether GFP-positive cells colocalize with pituitary hormones, we used a cocktail of antibodies against LHβ, FSHβ, PRL, TSHβ, ACTH, and GH (HORMONES, Fig 4). Colocalization of HORMONES with GFP-positive cells was not observed (Fig 4B–4Bʹʹʹ), while HORMONES/SOX2-double positive cells were present in the anterior (Fig 4B–4Bʹʹʹ, white arrowhead) and intermediate lobes (Fig 4C–4Cʹʹʹ, white arrowhead). In addition, GFP/SOX2-double positive cells were present in the intermediate lobes (Fig 4C–4Cʹʹʹ, yellow open arrowhead).

### Characterization of non-endocrine GFP-positive cells

Several studies to date have postulated that extrapituitary cells, which are non-pituitary in origin, such as neural and mesenchymal stem/progenitor cells and vessel precursor cells, are present in the pituitary gland [18–20]. GFP-positive cells can be clearly seen in Atwell’s recess but not in the anterior pituitary at E15.5 (Fig 1B). To further characterize GFP-positive cells, histochemical analysis using several cell markers was performed as follows.

**Fig 5:**
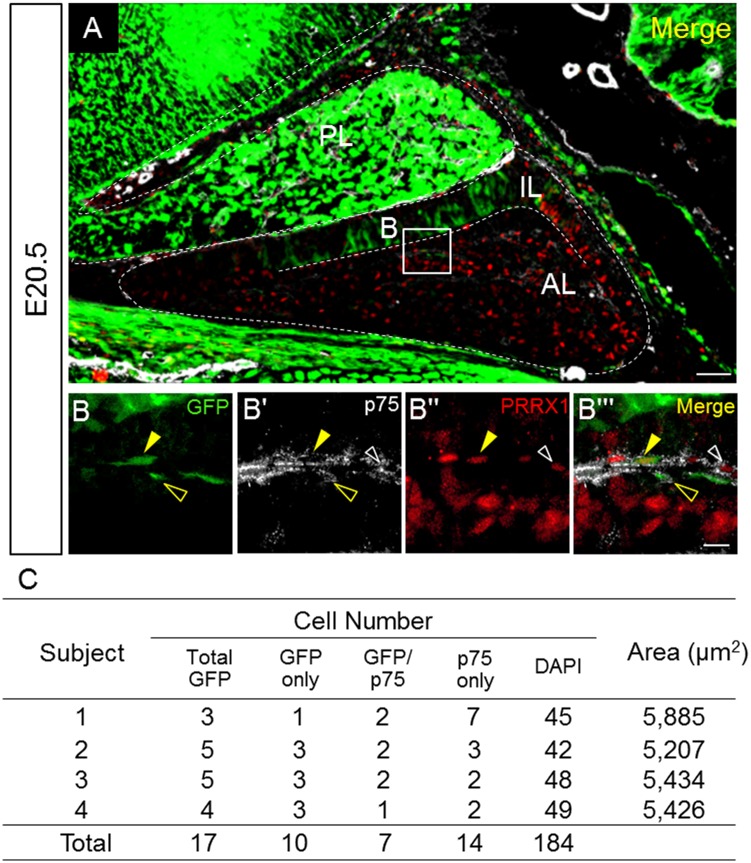
Triple immunostaining for GFP, p75, and PRRX1. Triple immunostaining for GFP (*green*), the neural crest marker p75 (*white*), and PRRX1 (*red*) was performed on E20.5. The boxed area in **A** is enlarged in **B–Bʹʹʹ**. GFP/p75/PRRX1-triple (*yellow arrowheads*), GFP/p75-double (*yellow open arrowheads*), and p75/PRRX1-double (*white open arrowheads*) positive cells are indicated. Counts of each cell type (n = 2, with two slices each) is listed in **C**. *AL* anterior lobe; *IL* intermediate lobe; *PL* posterior lobe. Bars = 50 μm (**A**) and10 μm (**B–Bʹʹʹ**).

First, as indicated in Fig 5, immunohistochemistry for p75, a neural crest cell marker [21], together with staining for GFP and PRRX1 showed the presence of GFP/p75/PRRX1-triple and GFP/Ki67-double positive cells, as well as p75/PRRX1-double positive cells. Notably, the p75-positive cells were elongated in shape (Fig 5B–5Bʹʹʹ). Results revealed that 41.2% of GFP-positive cells were GFP/Ki67-double positive cells (Fig 5C).

**Fig 6:**
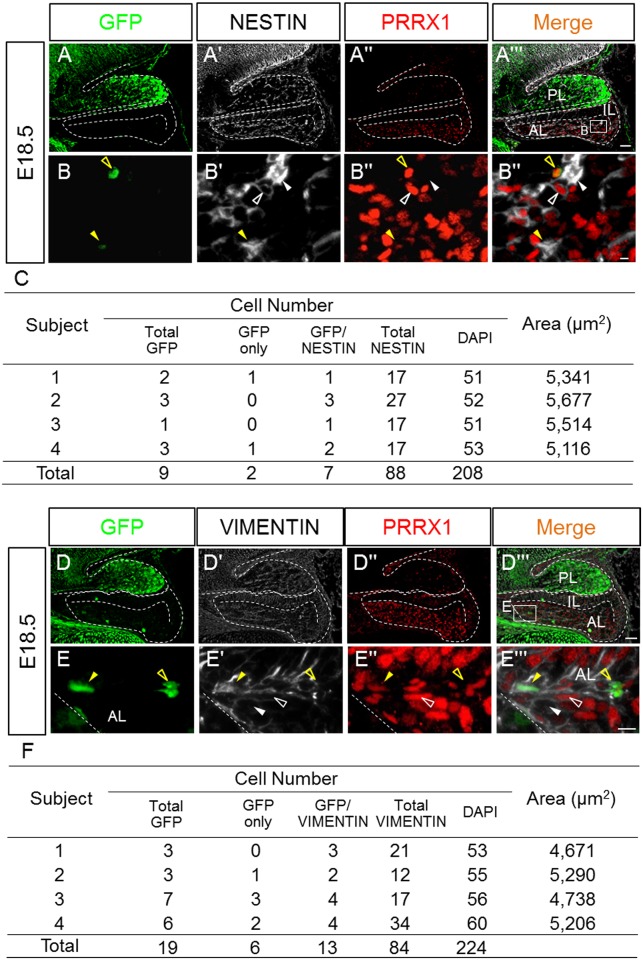
Triple immunostaining for GFP, PRRX1, and NESTIN or VIMENTIN. Triple immunostaining for GFP (*green*), PRRX1 (*red*), and NESTIN or VIMENTIN (*white*) was performed on E18.5. Merged images are shown on the right. The boxed areas in **Aʹʹʹ** and **Dʹʹʹ** are enlarged in **B–B‴** and **E-E‴**, respectively. GFP/PRRX1/NESTIN- and GFP/PRRX1/VIMENTIN-triple positive, GFP/PRRX1- and GFP/VIMENTIN-double positive, PRRX1/NESTIN- and PRRX1/VIMENTIN-double positive, and NESTIN- or VIMENTIN-single positive cells are indicated. Each cell type was counted, and the results are listed in **C** (n = 2 with two slices each) and **F** (n = 1 with four slices). *AL* anterior lobe; *IL* intermediate lobe; *PL* posterior lobe. Bars = 50 μm (**Aʹʹʹ** and **Dʹʹʹ**) and 10 μm (**Bʹʹʹ** and **Eʹʹʹ**).

Immunohistochemical analysis of NESTIN, a neural and mesenchymal stem/progenitor cell marker [22–25], together with GFP and PRRX1 staining revealed the presence of GFP/NESTIN/PRRX1-triple positive and GFP/PRRX1-double positive cells (Fig 6). Approximately 8.0% of the NESTIN-positive cells were also positive for GFP (Fig 6C). Then, VIMENTIN, a mesenchymal progenitor cell marker [26], was immunostained together with GFP and PRRX1. The results revealed a heterogeneous population of VIMENTIN-positive cells, with GFP/VIMENTIN/PRRX1-triple positive cells, VIMENTIN/PRRX1-double positive cells, and VIMENTIN-single positive cells (Fig 6E–6Eʹʹʹ). Approximately 15.5% of the VIMENTIN-positive cells were also positive to GFP (Fig 6F).

**Fig 7:**
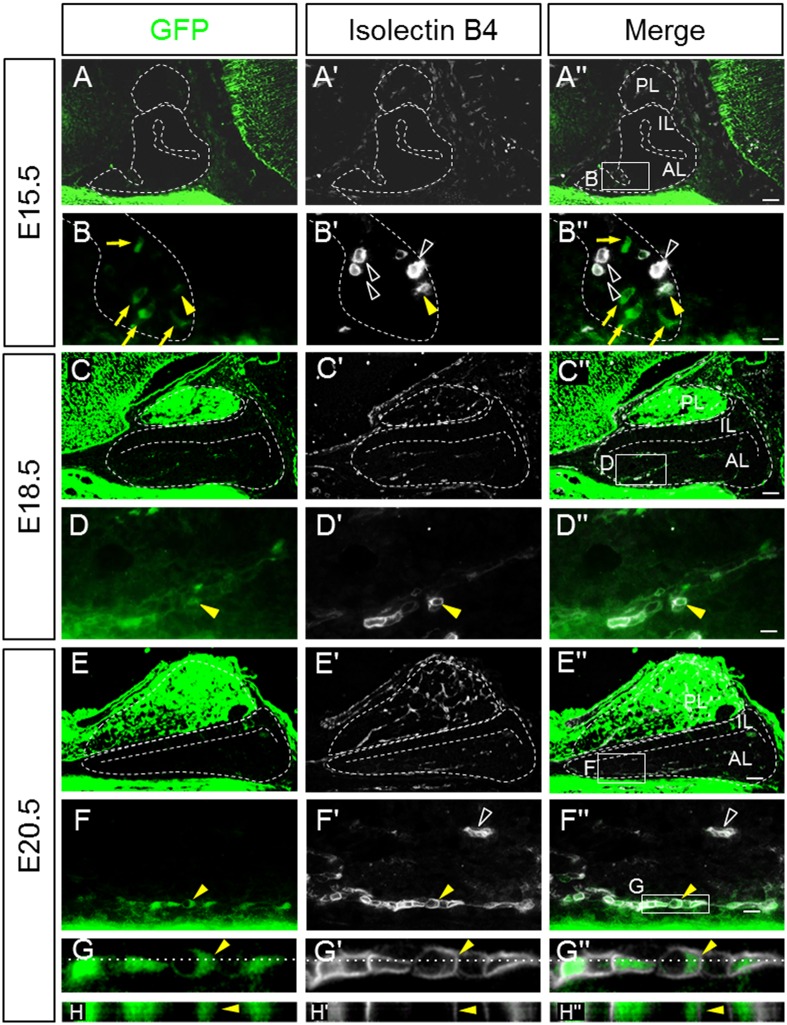
Double immunostaining of GFP and isolectin B4. Using sagittal sections of embryonic pituitaries on E15.5 (**A–Bʹʹ**), E18.5 (**C–Dʹʹ**), and E20.5 (**E–Hʹʹ**), immunostaining was performed on four slices for GFP and endothelial cell marker isolectin B4 and was visualized with FITC (*green*) and Cy5 (*white*), respectively. Merged images are shown on the right. The boxed areas in **Aʹʹ**, **Cʹʹ**, and **Eʹʹ** are enlarged in **B**–**Bʹʹ**, **D**–**Dʹʹ**, and **F**–**Fʹʹ**. GFP/isolectin B4-double (*yellow arrowheads*), GFP-single (*yellow arrows*), and isolectin B4-single (*open white arrowheads*) cells are indicated. The boxed area in **Fʹʹ** is enlarged in **G–Gʹʹ**, and its orthogonal projections were analyzed by confocal Z-stack imaging with 0.1-μm slices (**H**–**Hʹʹ**). *AL* anterior lobe; *IL* intermediate lobe; *PL* posterior lobe. Bars = 50 μm (**Aʹʹ**, **Cʹʹ**, **Eʹʹ**, and **Gʹʹ**) and 10 μm (**Bʹʹ**, **Dʹʹ**, **Fʹʹ**, and **Hʹʹ**).

More recently, we have demonstrated that PRRX1-positive mesenchymal cells invade through Atwell's recess during pituitary vasculogenesis [18,19]. To further verify the correlation between GFP-positive cells and blood vessels, we performed a histochemical analysis with fluorescence-labeled isolectin B4, a marker of vascular endothelial cells. A few isolectin B4-positive cells were observed in Atwell’s recess and the region surrounding the pituitary gland, but none were observed in the anterior lobe on E15.5 (Fig 7). Notably, GFP/isolectin B4-double positive cells were present at Atwell's recess, in addition to GFP-single and isolectin B4-single positive cells (Fig 7B–7Bʹʹ). GFP-positive and isolectin B4-positive cells were visible in the parenchyma of the anterior pituitary on E18.5 (Fig 7D–7Dʹʹ). GFP-positive cells were likely to enter into the anterior lobe, and some were positive for isolectin B4. Each cell type was then counted (Table 1). The frequency of GFP/isolectin B4-double positive cells in the parenchyma of the anterior lobe on E18.5 (9/26, 34.6%) was slightly higher than that in Atwell’s recess on E15.5 (6/38, 15.8%). In contrast, the frequency of total isolectin B4-positive cells decreased, from 18.0% (38/208) in Atwell’s recess on E15.5 to 10.6% (26/246) in the parenchyma of the anterior lobe on E18.5 (Table 1), reflecting the progress of cell differentiation and scattering in the parenchyma.

**Table 1:** The frequency of GFP/isolectin B4-double positive cells on E15.5 and 18.5.

A. Atwell’s recess on E15.5						
Subject		Cell Number				Area (μm2)
	Total GFP	GFP only	GFP/ Isolectin B4	Total Isolectin B4	DAPI	
1	4	3	1	8	49	4,671
2	4	3	1	5	47	4,187
3	3	2	1	13	56	4,708
4	5	2	3	12	56	4,617
Total	16	10	6	38	208	
B. Parenchyma on E18.5						
Subject		Cell Number				Area (μm2)
	Total GFP	GFP only	GFP/ Isolectin B4	Total Isolectin B4	DAPI	
1	3	1	2	7	56	5,608
2	5	2	3	6	54	5,579
3	4	1	2	5	67	4,943
4	3	1	2	8	69	5,137
Total	15	5	9	26	246	

Cell counts represent cells positive for GFP and /or isolectin B4.

**Fig 8:**
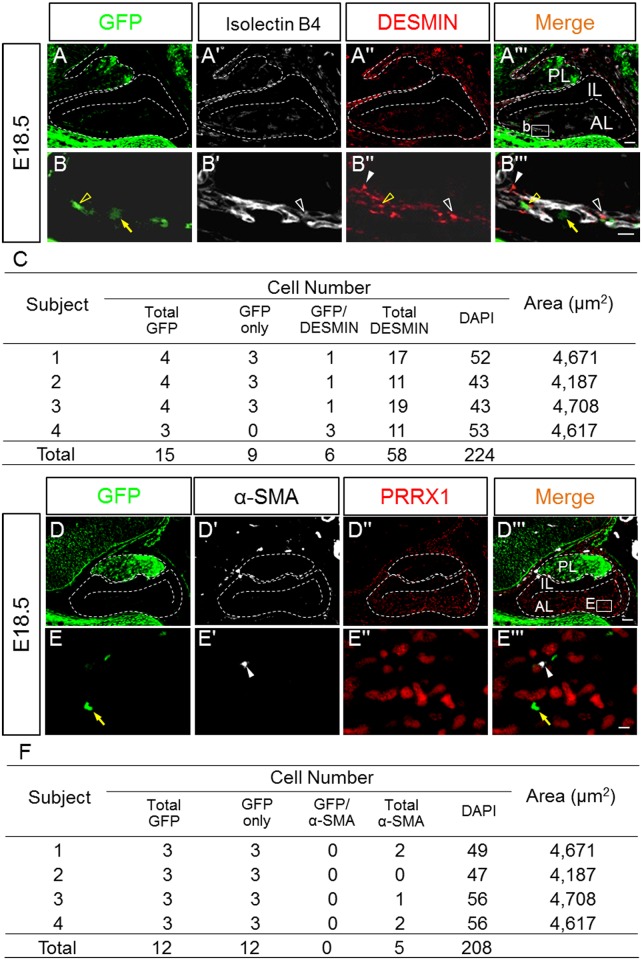
Immunostaining for GFP, DESMIN, α-SMA and PRRX1 and staining with isolectin B4. Using sagittal sections of embryonic pituitaries on E18.5, staining for vascular endothelial cells with isolectin B4 (**Aʹ**) or α-SMA (**Dʹ**) was performed by visualization with Cy5 (*white*), together with staining of GFP (FITC; *green*, **A** and **B**) and the pericyte marker DESMIN or PRRX1 (Cy3; *red*, **Aʹʹ** and **Bʹʹ**). GFP/DESMIN-double (*yellow open arrowheads*), DESMIN/isolectin B4-double (*white open arrowheads*), GFP-single (*yellow arrows*), and DESMIN- or α-SMA-single (*white arrowheads*) positive cells are indicated. Merged images (**Aʹʹʹ**, **Bʹʹʹ**, **Dʹʹʹ**, **and Eʹʹʹ**) are shown on the right. Each cell type (n = 2 with two slices each) was counted, and results are listed in **C** and **E**. *AL* anterior lobe; *IL* intermediate lobe; *PL* posterior lobe. Bars = 50 μm (**Aʹʹʹ** and **Dʹʹʹ**) and 10 μm (**Bʹʹʹ** and **Eʹʹʹ**).

Immunohistochemical analysis of DESMIN, a marker of immature and mature pericytes [27], was performed together with analysis of GFP and isolectin B4 (Fig 8). DESMIN-positive cells were observed along with isolectin B4-positive cells, some of which colocalized with GFP. In addition, DESMIN/isolectin B4-double and DESMIN-single positive cells were observed. GFP-positive cells were also positive for DESMIN at a frequency of 40.0% (6/15) (Fig 8C). Finally, immunohistochemical analysis of α-SMA, an early vascular marker present in vascular smooth muscle cells and pericytes [28], was performed. As shown in Fig 8D and 8E, a small number of α-SMA-positive cells was observed and were negative for GFP. PRRX1-positive cells were negative for α-SMA.

## Discussion

S100β-positive cells play special roles as non-endocrine cells in the anterior lobe of the pituitary gland. In the present study, we examined how S100β-positive cells arrive in the embryonic pituitary. Thus, we have demonstrated for the first time that extrapituitary S100β-positive cells exhibit diverse characteristics, such as those typical of vascular cells, mesenchymal cells, and neural crest cells. They also exhibit proliferation activity and invade the embryonic anterior lobe of the pituitary gland.

The S100β protein is often used as a tumor marker, and it is believed to exhibit diverse biological functions [29–31]. S100β has attracted attention owing to its characteristic presence in non-endocrine cells involved in various pituitary functions. Since the first observation of S100β in the anterior pituitary [32], many studies have suggested that S100β-positive cells play several distinct roles, such as being involved in phagocytosis, cell-cell communication, hormone release, and the maintenance of cell resources as stem/progenitor cells [5–7]. S100β-positive cells in the anterior pituitary can be grouped into three main types: astrocyte-like cells expressing glial fibrillary acidic protein and/or vimentin [33], epithelial cell-like cells expressing keratin [34], and dendritic cell-like cells expressing interleukin-6 [9,35–37]. This might suggest the presence of heterogeneous lineages of S100β-positive cells. Recently, we showed that a subset of S100β-positive cells has the ability to differentiate into hormone-producing cells [12,13], consistent with previous indications [38]. The generation of S100β-positive cells from SOX2-positive cells has been demonstrated using genetic lineage tracing [39]. These studies were conducted with postnatal pituitaries, as it was believed that S100β-positive cells appear approximately ten days after birth [15]. However, our previous study revealed the presence of *S100β* transcripts in the embryonic pituitary [8], indicating that S100β-positive cells are already present in the embryonic pituitary. Here, we demonstrated that S100β-positive cells at Atwell's recess and in the embryonic anterior lobe are SOX2-negative, differing from SOX2-lineage S100β-positive cells [39]. These appear by extrapituitary invasion with other mesenchymal cells.

The oral ectoderm, a pituitary primordium, originates from the thickened epithelium of an early neural primordium, the cranial placode of neural plate origin [40,41]. However, our recent results suggest that non-neural-plate originating cells positive for PRRX1 and PRRX2 appear in this tissue during organogenesis [18,19,42]. PRRX1 (also known as MHox) and PRRX2 (also known as S8) are known as mesenchymal markers and modulate, as well as act as, stem/progenitor cells [43–45]. We previously suggested that mesenchymal cells positive for PRRX1, PRRX2, and NESTIN are involved in pituitary vasculogenesis [18,19]. In the present study, we observed that S100β-positive cells are first negative for PRRX1 at Atwell's recess but are later positive for it in the anterior lobe, exhibiting transdifferentiation. Notably, Krylyshkina et al. [24] reported that some NESTIN-positive cells exhibit pericyte phenotypes and are sporadically positive for S100, exhibiting progenitor characteristics. Some S100β-positive cells were positive for NESTIN or VIMENTIN, which are known to indicate plasticity. Indeed, S100β/PRRX1-positive and S100β-positive cells are similar to vascular cells that are isolectin B4- and DESMIN-positive. However, S100β-positive cells are negative for α-SMA, indicating that a different cell lineage is responsible for generating smooth muscle cells. Accordingly, some S100β-positive cells may participate in vasculogenesis by transdifferentiation.

In the present study, we observed that some PRRX1- and S100β-positive cells are also positive for p75, exhibiting an elongated cell shape similar in appearance to vessels differentiating into pericytes and smooth muscle cells in the anterior lobe (Fig 5). p75 is a receptor for neurotrophin and is known as a neural crest marker [46]. Two decades ago, Borson et al. (1994) reported comparative data that showed that p75-positive cells are present in surrounding mesenchymal cells and blood vessels in the developing macaque pituitary [47]. These observations provide intriguing and suggestive insights for understanding pituitary organogenesis, since the neural crest, now referred to as the fourth germ layer in vertebrates, originates from the border area between the neural plate and non-neural ectoderm. This is followed by delamination and an epithelial-mesenchymal transition (EMT) to produce diverse cell lineage derivatives of the neural crest that then invade several tissues during the embryonic period [48–50]. These derivative lineages include pericytes, smooth muscle cells [51] and S100β-positive cells [52,53], the latter of which were observed in the present study. More recently, the involvement of neural crest cells in pituitary vasculogenesis has been reported [54]. Motohashi et al. (2014) revealed that neural crest-derived cells sustain their multipotency even after entry into their target tissues [55]. It should also be mentioned that the reverse transition from mesenchyme to epithelium includes the acquisition of stemness [56] and that neural crest-derived Schwann cells can be reprogrammed to acquire multipotency [57]. The role of neural crest lineage cells and their plasticity in the anterior lobe remain interesting subjects of study.

We previously showed that various types of cells invade into the pituitary gland, in particular S100β-positive cells with differentiation and proliferation abilities [19], confirming and exploring in further detail the previous results that extrapituitary lineage cells invade the anterior lobe [58]. A previous study that revealed the importance of direct contact between the pituitary primordium and surrounding ventral diencephalon, mesenchyme tissue, and notochord [58] suggested the partial invasion of surrounding cells, in addition to signals promoting growth and differentiation. In future studies, we intend to investigate whether S100β-positive and other extrapituitary cells maintain their plasticity and/or acquire stemness in the anterior lobe. To accomplish this, lineage tracing of S100β-positive cells will be required.

## Supporting Information

S1 Fig Triple-immunostaining for GFP, PRRX2, and SOX2. Triple-immunostaining for GFP (*green*), PRRX2 (*red*), and SOX2 (*white*) is shown in sections on E20.5. Merged images are shown on the right. The boxed area in **A**ʹʹʹ is enlarged in **B**–**B**ʹʹʹ. GFP/PRRX2-double positive (*yellow arrowheads*), GFP-single positive (*yellow arrows*) and PRRX2-single positive (*white arrowheads*) cells are indicated. *IL* intermediate lobe; *PL* posterior lobe. Bars = 50 μm (**Aʹʹʹ**) and 10 μm (**Bʹʹʹ**).
(TIF)
