# Supplementary figures and images for: S100β-Positive Cells of Mesenchymal Origin Reside in the Anterior Lobe of the Embryonic Pituitary Gland
Source: PLoS One. 2016 Oct 3;11(10):e0163981. doi: 10.1371/journal.pone.0163981 (PMC5047643; doi:10.1371/journal.pone.0163981)

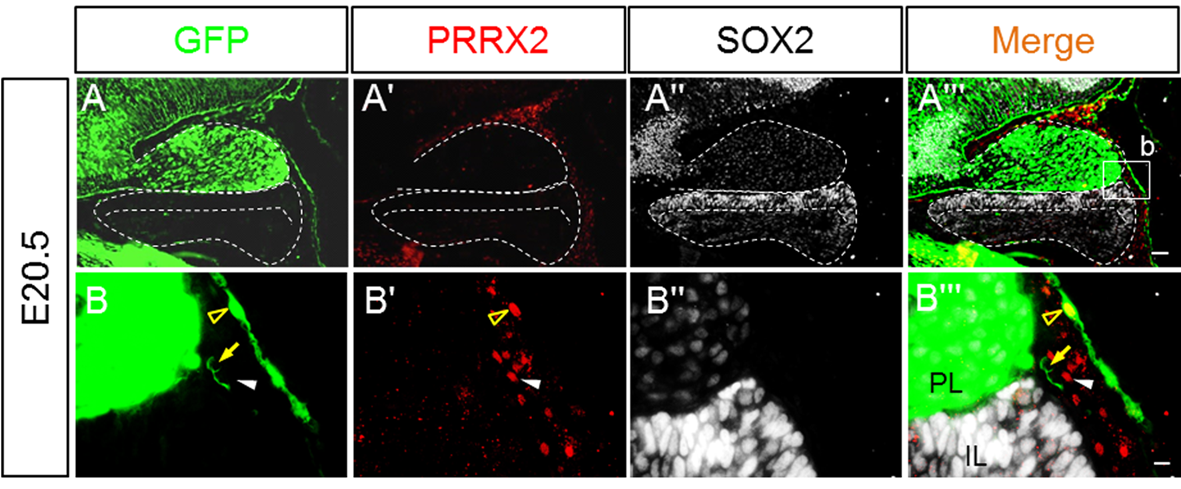

Supplement: S1 Fig — Triple-immunostaining for GFP (green), PRRX2 (red), and SOX2 (white) is shown in sections on E20.5. Merged images are shown on the right. The boxed area in Aʹʹʹ is enlarged in B–Bʹʹʹ. GFP/PRRX2-double positive (yellow arrowheads), GFP-single positive (yellow arrows) and PRRX2-single positive (white arrowheads) cells are indicated. IL intermediate lobe; PL posterior lobe. Bars = 50 μm (Aʹʹʹ) and 10 μm (Bʹʹʹ). (TIF) [file pone.0163981.s001.tif]
